# Feasibility of a national open data policy in Zimbabwe

**DOI:** 10.3389/frma.2022.985999

**Published:** 2022-08-12

**Authors:** Josiline Chigwada

**Affiliations:** Library Department, Chinhoyi University of Technology, Chinhoyi, Zimbabwe

**Keywords:** open government data (OGD), open data (OD), OECD open data initiatives, open data policy, open data barometer, ZIMSTAT, research data management (RDM)

## Abstract

A study on the feasibility of a national open data policy in Zimbabwe was done to document open government data globally and in Zimbabwe. The study showcases the benefits of open government data and the opportunities and challenges toward the development of a national open data policy. Web content analysis and document analysis were used to collect data concerning the readiness of the country in implementing open data activities. The open data barometer was used to gather qualitative data which is essential in assessing the preparedness of the country in opening up government and research data. Content analysis was used to analyse the data which was presented thematically based on the objectives of the study. The findings indicated that the Government of Zimbabwe has endorsed a couple of open data frameworks though some projects are done by non-governmental organizations. The major challenge is implementation of these conventions and commitment to make the data accessible. The results indicated that open data must be made available and accessible within Zimbabwe as a matter of national policy. The author recommends the need for advocacy and continuous awareness creation among the stakeholders so that a national open data policy can be crafted and enacted. The enactment of a national open data policy would guide the use of and access to government data and research data which is valuable in research.

## Introduction

Open Government Data (OGD) initiatives started in mid-2000 at central and local government levels whereby open government data portals were developed in Organisation of Economic Cooperation and Development (OECD) and non OECD countries (Ubaldi, 2013). This is a movement to open up government data by putting it into the public domain to create good conditions for social inclusivity and democracy. There is also a possibility of economic growth through the creation of new products and services using public data by individuals, private enterprises and civil society organizations. The advocates of open government data focus on data in government databases and they deal with the legal and technical issues of access, use and reuse of the databases. The government creates a lot of data through the various ministries as they conduct their day to day activities and this data is valuable to researchers and decision makers as a way of solving the challenges that are being faced in various countries. In Zimbabwe, the concept of opening up government data was spearheaded by non-governmental organizations and the Open Data Inventory (ODIN) ranks Zimbabwe at number 97 in 2020 with an overall score of 48 (Open Data Watch, 2020). In order for government data to be accessible and useful, there is need for policy backup and this calls the need for a national open data policy in Zimbabwe. It is against this background that the study seeks to answer the following objectives:

- To examine the open data landscape in Zimbabwe.
- To analyze open government and open government data in Zimbabwe.
- To identify the opportunities and challenges that can be used to inform the open data policy for the country.

### Problem statement

The open government data movement is concerned with the release of large amounts of government data in various formats and conditions that allow re-use of the data. The development of open science in Zimbabwe is still in its infancy stage as pointed out by Chigwada et al. (2017). The study revealed that there is no open data repository in Zimbabwe and researchers do not want to share their data in fear of intellectual property rights and data abuse. Chiparausha and Chigwada (2019) concurred and added that data is not accessible in Zimbabwe. Government ministries are not willing to share their data because of confidentiality issues and lack of trust among the researchers in fear of abuse of the data. This can also be worsened by the existence of harsh legislation such as the Access to Information and Protection of Privacy Act (AIPPA) of 2002 and Public Order and Security Act (POSA) which could negatively impact open data and information dissemination in Zimbabwe. During the open data day commemorations in March 2019, researchers lamented over the inaccessibility of government data in Zimbabwe where they pointed out that one has to pay large sums of money to access data for research purposes (Open Knowledge Foundation, 2019). The current economic situation in Zimbabwe can also affect the development of open government data initiatives in Zimbabwe since people cannot purchase modern software and equipment to improve the digitization processes. There are also political factors that affect the proper dissemination of information and data which allow some centers to hide damaging government data, and the lack of accountability in data sharing leading to some institutions not releasing nor accounting for their data. The National Open Data Policy can assist in solving some of these challenges. It is against this background that a study on the feasibility of a national policy on open data in Zimbabwe was done to showcase the open data landscape in Zimbabwe pointing out the opportunities and challenges that can be used to inform policy development in the country. This is a background study that was done which would be followed by other studies which would strategise how the policy can be enacted and implemented and how awareness can be created among the policy makers and the general populace. The open data subject is still new in the country meaning that a lot of awareness creation is essential to conscientise the stakeholders on the need and the importance of a national open data policy in Zimbabwe.

## Literature review

### Open government and open government data globally

Advocates in United States of America (USA) published a set of open government data principles in 2008 leading to the advent of the term Open Government Data (OGD) (Laboutkova, 2015; Hodess, 2019; Tauberer, 2022). The term can be easily understood by defining government data and open data. Government data is regarded as data that is produced by public bodies by using public funds collected through taxes while open data is the data that can be used, reused, distributed freely (OECD, 2022). Open government data initiatives include budget information, business information, registers, patent, and trademark information, public tender databases, geographic information, legal information, meteorological information, social data, and transport information (Ubaldi, 2013; Attard et al., 2015; OECD, 2022). There is a lot of data which is produced by public institutions and opening up this data increases the transparency and accountability to citizens. There is a project called OECD open government data which has a mandate of progressing international efforts on open government data impact assessment (OECD, 2022). Therefore, the open government data principles stipulate that government data shall be considered open if it is complete, primary, timely, accessible, machine processable, non-discriminatory, non-proprietary, and license-free (Tauberer, 2014; Gong, 2016). The Sunlight Foundation added two more principles on top of the eight which are permanence and usage costs (Ubaldi, 2013). It has been stated that African countries are now opening up their data to attain transparency and accountability. However, although the data portals are being created, there is need for data provisioning in the open formats to populate the portals (Bello et al., 2016). This shows that in both the developed and developing nations, the knowledge of the principles of open government and open government data are known but the challenge is on implementing them.

### Open government and open government data in Zimbabwe

In Zimbabwe, although the data is available, it is not accessible as pointed out by researchers who attended the open data day commemorations at Bindura University of Science Education Library (Open Knowledge Foundation, 2019). Although there have been some baby steps toward the implementation of OECD open government data initiatives in Zimbabwe, the data is only available as summaries (The World Bank, 2019). The open data barometer (Web Foundation, 2018c) which is a global measure indicating how governments are publishing and using open data for accountability, innovation and social impact is one of the measurements that can be used to assess if Zimbabwe is moving along with the current trends in open government data. The open data barometer is also regarded as a research tool that is used to measure the prevalence and impact of open data initiatives in governments around the world (Web Foundation, 2018a).

### Beneficiaries of open government data

Open government data is meant to benefit all the stakeholders that are involved which are the government, citizens, civil society and the wider economy including the private sector (Ubaldi, 2013; Gonzalez-Zapata and Heeks, 2015; Pereira et al., 2017; Open Data Govlab, 2019; Open Government Partnership, 2019; Zuiderwijk et al., 2019). The government would be able to make strategic decisions using the data and the data would provide ways of conducting business and allocating resources to be efficient and effective in delivering services. The data would assist in dealing with fraud and other corruption challenges being faced by the government and smarter and innovative services would be delivered to the public. This would also improve the way the government interacts with the users. Open government data help in offering services in a transparent way (OECD, 2022). Citizens benefit through public participation and social engagement in addressing the needs of the public. Therefore, open government data help citizens to increase their quality of life. Civil society organizations play a major role in working with vulnerable segments of the population, e.g., the Sunlight Foundation and the Open Forum Foundation in the United States of America, the Open Knowledge Foundation in Germany and the Open Rights Foundation in the United Kingdom. The private sector is one of the beneficiaries of open government data since they can use the datasets for commercial purposes (Open Government Partnership, 2019). This would assist in providing innovation and experimentation in service delivery. However, these can be regarded as competitors by the government and this can contribute to the reasons why the government institutions do not want to make their data open. However, from the general public and researchers' perspectives, it is difficult to derive these benefits since the data is not easily accessible (Open Knowledge Foundation, 2019).

### Benefits of open government and open government data

Open government data is important in increasing public transparency where the public is able to understand what is being done by the government and how well it is performing (The World Bank, 2019). This helps in holding the government accountable for any wrongdoings or if it fails to achieve the results it would have set. It is also a way of showing the good things that the government would be doing since it is accountable to the general population in service delivery. Opening government data helps to generate insights on how to improve government performance through public participation and collaboration in creating value added services (The World Bank, 2019). This shows that individuals and governments would improve the decision making process whereby the public uses government data to have better decisions that improve the quality of life. As a result, the collaborative effort in decision making would ensure that there is trust between the service providers and those who are at the receiving end. Open government data is viewed as a source of economic growth, social innovation and new forms of entrepreneurships (Ubaldi, 2013). The goals of open government data in addition to higher transparency and public accountability is innovation, efficiency and flexibility in government (Yu and Robinson, 2012). Innovation calls for the introduction of new services that are beneficial to the nation leading to less resistance if changes are introduced. The Web Foundation (2018a) summarized the benefits of open data as improving how government resources are used, driving more transparency, accountability and participation, driving social impact by making the policy process more inclusive, and having positive economic impact by boosting economic growth. However, from the Zimbabwean perspective, the custodian of the data is the only stakeholder that is fully benefiting from such data since only summaries are available for public consumption.

### Challenges against open data policy

The major challenges in regard to open government data include privacy, legal, financial and technological issues (Ubaldi, 2013). When dealing with data, it needs to be relevant, easily accessible, and reusable by everyone. As a result, there is need to protect the privacy of individuals and ensure that the data can be easily reused. The government should get feedback from data users to check if the data is useful, relevant and accessible to work toward ensuring openness, transparency, accountability, sharing, collaborating and public engagement (Ubaldi, 2013). The FAIR data principles also call for the findable, accessible, interoperable and reusable of data and the principles provide guidance for data management and stewardship (Wilkinson et al., 2016; Liber, 2017).

## Materials and methods

The study was qualitative in nature whereby data was collected from websites and documents mainly using the parameters of the open data barometer. Purposive sampling was used to select websites that document open data readiness and adoption so as to find out the stakeholders that should be involved in enacting and implementing a national open data policy. Web content analysis was used to visit government websites to confirm if the government data is now open. The open data barometer was also consulted to showcase where Zimbabwe is in terms of open data access and usage. Document analysis, looking at country reports and open data initiatives reports, was used to gather data on the infrastructure that is available and the opportunities that can be used to formulate a national policy on open data. Content analysis was used to analyse the results and they were presented thematically using the objectives of the study.

## Results and discussion

Findings from the web content analysis reveal that there is no open data policy in place in Zimbabwe and it is not a member of the Open Government Partnerships (Murape, 2021). However, the government had endorsed a couple of open data frameworks such as the Zimbabwe Open Data Initiative (ZODI), a project of the Institute of Data Science Management, Data Management Society, and The Pan African Business Council that works with companies, civil society, and government to build an open and trustworthy data ecosystem (Zimbabwe Open Data Initiative, 2022). Small steps had also been registered through Zimbabwe National Statistics Agency (ZimStat, 2021). The major challenge is implementation and domestication of these conventions so that they can benefit people as stated by Wilkinson et al. (2016) and Liber (2017). The open data landscape in Zimbabwe showed that the data is available but not accessible since every ministry produced its own data and most of the data is available as summaries as stated by The World Bank (2019). However, most of the data is in print format and accessing such data online is not possible. Efforts are being made to digitize such datasets and of note is the Zimbabwe data portal on Open Data for Africa platform (African Development Bank, 2022), although most of the data is not current. Zimbabwe introduced the concept of data centers and the national data center was commissioned in February 2021 (Myles, 2021; Swinhoe, 2021; Towindo, 2021) and would be used to centralize and digitize government services. There is a Centre for High Performance Computing for big data analysis in Zimbabwe which is currently located at the University of Zimbabwe and is open to every researcher and can be fully utilized if open government data initiatives are fully adopted (ZCHPC, 2022). The findings revealed that there is an Academy of Science in Zimbabwe that can assist in opening government data.

The Ministry of Higher and Tertiary Education, Science and Technology Development launched Education 5.0 which is a new development thrust anchored on scientific innovations (Government of Zimbabwe, 2019) and the STEM education which concentrate on Science, Technology, Engineering and Mathematics. It stated that institutions of higher learning should deal with teaching, research, community services, innovation, and industrialization making use of the disruptive technologies such as Internet of Things (IoT), advanced robotics, and automation of knowledge. This led to the development of innovation hubs at six universities to provide platforms for innovators and researchers to develop their innovations with the aid of those with technical and entrepreneurial skills (Satumba, 2021). Universities with complete innovation hubs are National University of Science and Technology, Midlands State University, University of Zimbabwe, Harare Institute of Technology, Zimbabwe Defence University, and the Chinhoyi University of Technology (Nyikadzino, 2022). All these institutions are public universities and if the data is open, it would benefit a lot of researchers and the general populace as stated by OECD (2022).

**Figure 1:**
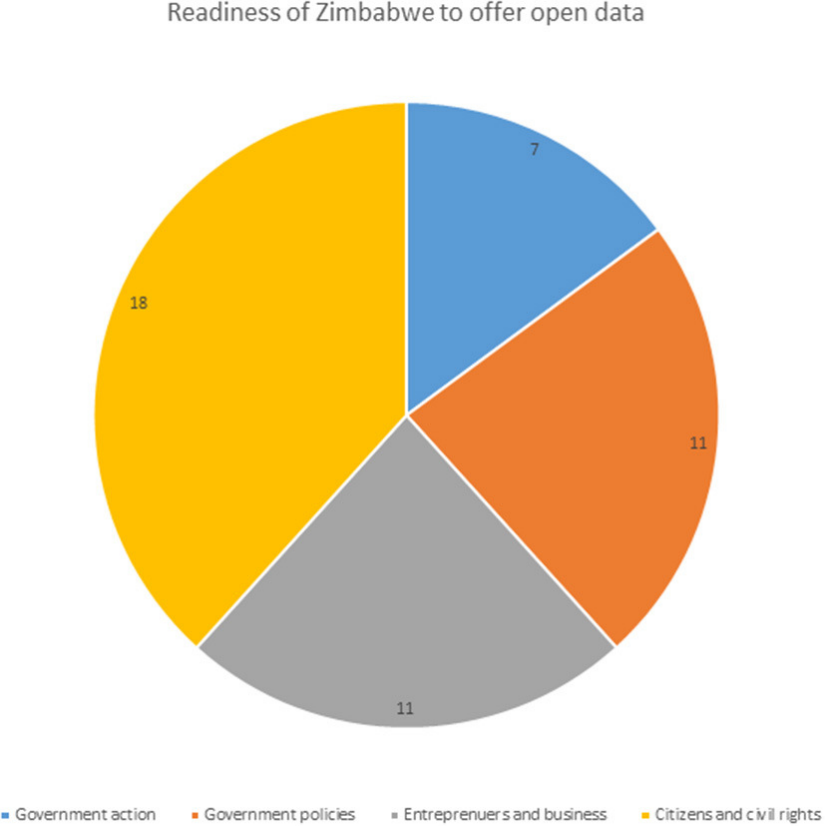
Readiness of Zimbabwe to offer open data–Data Source, Web Foundation (2018b).

A look at the open data barometer showed that there were 30 governments that have adopted the open data charter and have committed to G20 anti-corruption open data principles which are termed the leaders edition. The other 115 countries were just listed not as part of open data charter adopters in the year 2017 (Web Foundation, 2018c) and Zimbabwe is one of those 115 countries. This shows that there are signs that the Zimbabwean government is now moving toward open data initiatives but is not yet committed since it had not adopted the open data charter nor signed the G20 anti-corruption open data principles which are a globally agreed set of best practices for publishing, using and maximizing the potential of data (Web Foundation, 2018a). The barometer ranks governments on readiness for open data initiatives, implementation of open data programs, and impact that open data is having on business, politics, and civil society. There are three essential ingredients for good open data governance which are open by default, data infrastructure, and publishing with purpose. Open by default states that governments should build policies, skills and processes to enable a culture of data openness and acceptance of publishing open data. Data infrastructure deals with the building or improvement of technical infrastructure that supports openness in government and organizational transformation. Publishing with purpose is the consideration of the users of open data and what they use the data for to ensure that the data that is published is what is needed and the process is done in a way they can easily use the data (Web Foundation, 2018a). This buttresses what was stated by researchers during the open data day commemorations (Open Knowledge Foundation, 2019) who stated that they are not able to access the government data for research purposes. The readiness of the country to offer open government data is shown in Figure 1.

**Figure 2:**
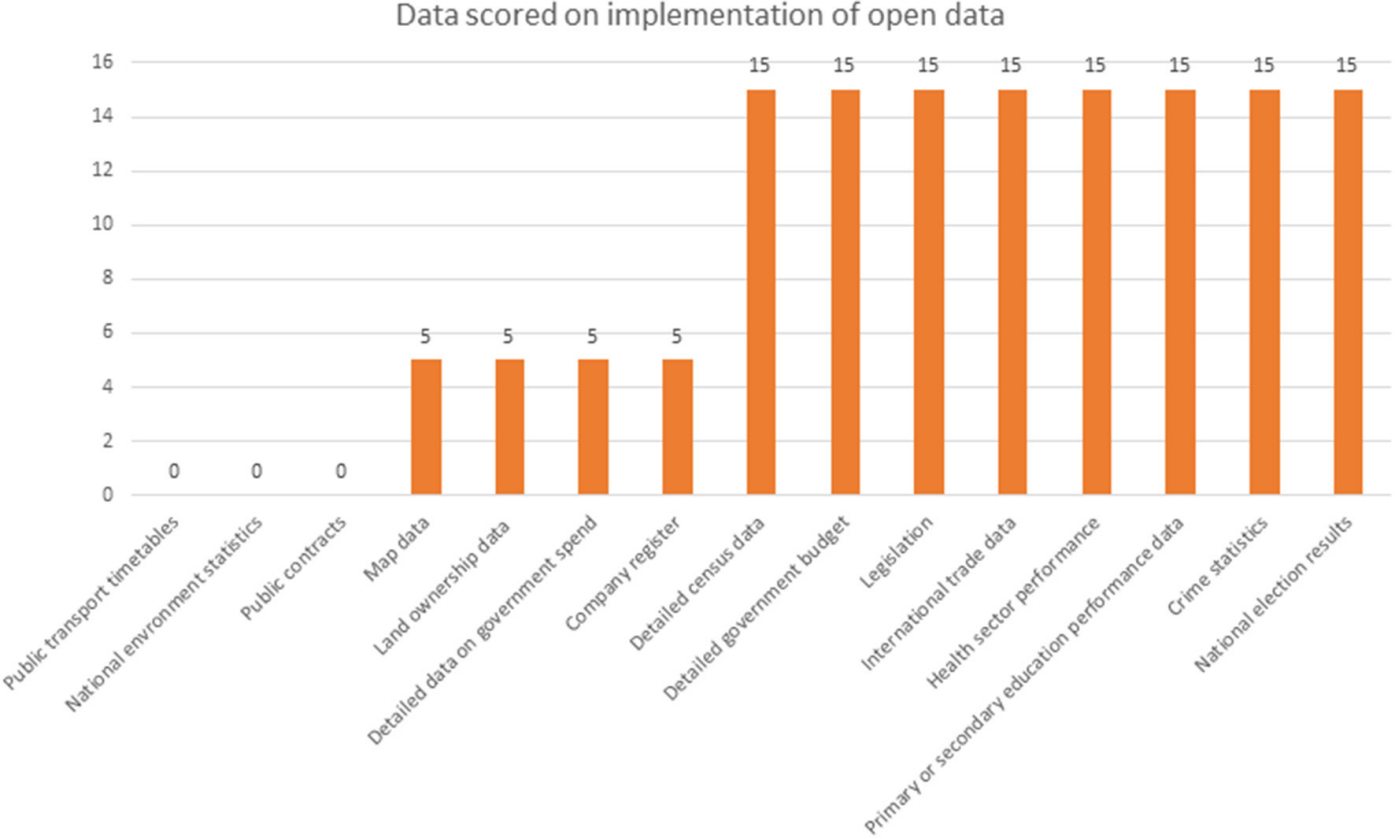
Status of Zimbabwe in implementation of open data–Data source, Web Foundation (2018b).

The summary of information about Zimbabwe on the open data barometer is shown in Figures 1, 2. The readiness status in Figure 1 showed that Zimbabwe scored 7 on government action, 11 on government policies, 11 on entrepreneurs and business and finally 18 on citizens and civil rights. This shows that all the sections of society should be involved in the process of moving toward open data as stated by Pereira et al. (2017), Open Data Govlab (2019), Open Government Partnership (2019), and Zuiderwijk et al. (2019).

Figure 2 showcases the datasets scored in various sectors toward the implementation of open data. A number of questions were asked which include does the data exist, is it available online from government in any form, is the dataset provided in machine-readable and reusable format, is the machine-readable and usable data available as a whole, is the dataset available free of charge, is the data openly licensed, is the dataset up to date, is the dataset being kept regularly updated, was it easy to find information about this dataset, and are data identifiers provided for key elements in the dataset? However, in terms of impact the country scored zero on political, social and economic impact of the available data.

**Figure 3:**
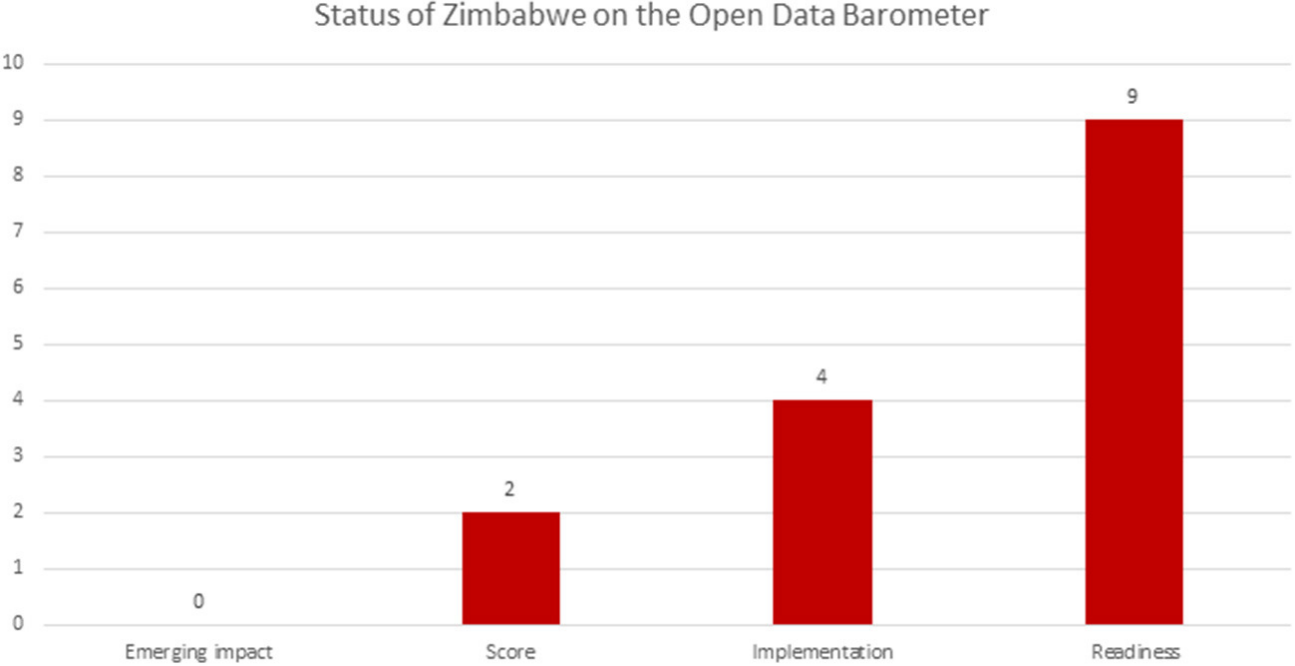
Status of Zimbabwe on the Open Data Barometer–Data Source, Web Foundation (2018c).

The status of Zimbabwe on the open data barometer 4th edition (Web Foundation, 2018c) showed that Zimbabwe is on number 112 with a score of 2 out of 100 as shown in Figure 3. The score is an overall assessment of the prevalence of open data initiatives. On readiness, Zimbabwe scored 9 out of 100 and this is regarded as the readiness of states, citizens, and entrepreneurs to secure the benefits of open data. On implementation, the score is 4 out of 100 showing the extent to which accessible, timely, and open data is published by each country government on a selection of 15 key fields. On emerging impact, the score is 0 out of 100. This is the extent to which there is any evidence that open data release by the country government has had the impacts in a variety of different domains in the country. As a result, the findings showed that there is currently no evidence on the use and impact of open government data in Zimbabwe and therefore it is difficult to enjoy the benefits of open government data that were pointed out by Yu and Robinson (2012); Ubaldi (2013); Web Foundation (2018a), and The World Bank (2019).

The results indicated that open data must be made available and accessible in Zimbabwe as a matter of national policy to ensure transparency, innovation and efficiency as stated by Yu and Robinson (2012) and Ubaldi (2013). This calls for the need for advocacy and continuous awareness creation among the stakeholders so that a national open data policy can be crafted and enacted. This can be achieved by taking into consideration the open government data principles pointed out by Ubaldi (2013), Tauberer (2014), Laboutkova (2015), Gong (2016), Hodess (2019), and Tauberer (2022).

## Conclusion and recommendations

In conclusion, it can be noted that a lot of government data is generated every day and there is need for a national open data policy that would clearly state what open data is and its benefits to the nation at large. It is feasible to have a national open data policy in Zimbabwe since there were some baby steps that were taken by ZimStat and the meteorological department to open up some data and this shows that the government is aware of the benefits of open data. The appearance of Zimbabwe on the open data barometer is a good indicator that there is open government and open government data in Zimbabwe though it is still minimal. The infrastructure that is needed to run the project is already available in Zimbabwe as evidenced by the commitment of various ministries in developing innovation hubs, and data centers which shows political will to make the data available. However, the major challenge that is faced is the accessibility of this data as well as the adoption of the open data charter so as to abide by the 10 principles of open government which are completeness, primacy, timeliness, ease of physical and electronic access, machine readability, non-discrimination, use of commonly owned standards, licensing, permanence and usage costs.

The author recommends the need to build capacity on open government data to ensure that researchers would benefit from the data that is collected using public funds, and to enhance technological and data skills among the researchers and librarians when dealing with data. The stakeholders should be knowledgeable about these principles to ensure that full benefits are drawn from the open government data. Government data should be open by law and it helps to achieve national development and subsequently attain vision 2030 as stated in the sustainable development goals in Zimbabwe, and make research more valuable to the nation. There is need to ensure that there is advocacy and continuous awareness creation among all the stakeholders that are involved so that no one is left behind. The stakeholders include academia, government, corporate world and other professional bodies. This would ensure that equipment, infrastructure, human, and financial resources needed for open data are available. The infrastructure should also be maintained and upgraded to enhance service delivery. The government must prioritize open data governance so that open data becomes part and parcel of how they run their day to day business. There is need for the development of plans, guidelines and procedures of opening up data. The African Open Science Platform (AOSP) had been working on data policy issues with guidance from the Committee on Data for Science and Technology (CODATA) and Zimbabwe can benefit from this initiative to enact and implement a national open data policy.

## Data availability statement

The raw data supporting the conclusions of this article will be made available by the authors, without undue reservation.

## Author contributions

The author confirms being the sole contributor of this work and has approved it for publication.

## Conflict of interest

The author declares that the research was conducted in the absence of any commercial or financial relationships that could be construed as a potential conflict of interest.

## Publisher's note

All claims expressed in this article are solely those of the authors and do not necessarily represent those of their affiliated organizations, or those of the publisher, the editors and the reviewers. Any product that may be evaluated in this article, or claim that may be made by its manufacturer, is not guaranteed or endorsed by the publisher.
